# A Dual-Layer Frequency Selective Surfaces with Tunable Transmission and Fixed Absorption Bands

**DOI:** 10.3390/ma18184414

**Published:** 2025-09-22

**Authors:** Zhiming Zhang, Qingyang Wang, Qiyuan Wang, Pei Liu, Yun He, Mingyu Li

**Affiliations:** 1 School of Information Engineering, Wuhan University of Technology, Wuhan 430070, China; zhangzhiming@whut.edu.cn (Z.Z.); 18973418795@163.com (Q.W.); w2729130943@163.com (Q.W.); pei.liu@whut.edu.cn (P.L.); 2 School of Physics and Mechanics, Wuhan University of Technology, Wuhan 430070, China

**Keywords:** frequency selective surface, tunable transmission, absorption

## Abstract

This paper presents dual-layer frequency selective surfaces (FSSs) with frequency division control function through an integrated tunable transmission window at a lower frequency and an absorption performance at a higher frequency. The bottom frequency selective surface (FSS) layer, configured as a bandpass structure, incorporates a gradient gap square-ring element loaded with varactor diodes. This configuration enables dynamic tuning of the L-band transmission window from 1.26 GHz to 1.9 GHz via varactor capacitance modulation. Simultaneously, the top FSS layer utilizes a square-ring-cross-slot topology. Leveraging the strong reflection characteristic of the bottom FSS at higher frequencies in conjunction with dielectric loss mechanisms, the structure achieves absorption performance within the 5.56 GHz to 5.72 GHz band. Measurement results indicate insertion loss at operational frequencies within the transmission window remains below 1.41 dB, while the absorption peak reaches approximately −30 dB. Close agreement between simulated and measured results validates the proposed design.

## 1. Introduction

With the rapid development of wireless communication and radar technologies, the demand for dynamic electromagnetic wave manipulation has significantly increased. Frequency selective surfaces, functioning as spatial filters, can control transmission or reflection characteristics at specific frequency bands through periodic structures [1], finding critical applications in communication antennas [2,3], electromagnetic shielding [4,5], and absorbers [6,7,8,9]. However, conventional FSSs exhibit fixed electromagnetic responses post fabrication, limiting their adaptability to dynamic reconfiguration in complex electromagnetic environments.

Recent advancements in tunable FSSs, integrating active components such as varactor diodes [10,11,12], PIN diodes [13], MEMS switches [14] and so on, have enabled dynamic adjustments of resonant frequencies or bandwidths. Tunable frequency selective surfaces (TFSSs) can be broadly categorized into two types: frequency-tunable FSSs [15,16] and function-switchable FSSs [17,18]. In frequency-tunable designs, varactor diodes are typically integrated into the active layer of absorptive FSSs to enable resonance frequency reconfiguration through bias voltage-controlled capacitance modulation. For instance, an electromagnetic transmission structure was realized by symmetrically embedding varactors in the interlayer gaps of cascaded bandpass FSSs [19]. The regulation performance of the working frequency band can be achieved by adjusting the excitation voltage of the varactor diode [16]. Similarly, PIN diodes have been leveraged for scattering parameter amplitude control through their voltage-dependent resistance characteristics [20], which can achieve the regulation and control of the absorbing frequency band.

Furthermore, another critical application of PIN diodes lies in TFSSs, where diode state modulation (ON/OFF) enables dynamic functionality switching. This capability has driven the development of frequency selective rasorbers [21,22,23] (FSRs) capable of transitioning between absorptive and transmissive modes through PIN diode control. For example, the switchable FSR could be tuned from passband into absorption band through PIN diode mode switching [24], and a similar method can also be applied to multi-layer wideband absorbing structures to achieve the switching of transmission and reflection performance within the band [25]. However, they focus on the adjustment of the working frequency band with single function or the functional switching in a fixed working frequency band. The comprehensive design of compound functions and the reconfiguration of the working frequency band still need further research.

In this paper, tunable dual-layer multi-functional FSSs are proposed to achieve a compatible design between the transmission reconfigurability and the invariance of the absorbing window. The tunable performance of transmission is achieved through the bottom tunable FSS layer based on the gradient gap square-ring loaded with varactor diodes. The absorption performance is obtained through the combination of the top FSS layer and the bottom tunable FSS layer. Moreover, the absorption performance is not affected by the regulation performance of the transmission window.

The structure of this article is as follows. In Section 2, we first introduce the overall architecture of the dual-layer FSSs, and then successively elaborate on the unit structure design process of the tunable transmission and the fixed absorption performance, including the analysis of the equivalent circuit. Based on this, the integrated architecture is designed and the simulation results are analyzed. In Section 3, the proposed dual-layer FSSs are experimentally verified and the test results are discussed. It can be found that there is good consistency between the measured results and the simulation results. Section 4 compares this work with previous related research. Finally, in Section 5, the content of the entire article is summarized.

## 2. Analysis and Design

This section presents the proposed dual-layer FSS architecture, detailing its dual-layer topology and unit cell designs for both upper and lower layers. In this process, the equivalent circuit is used to study the qualitative relationship between parameters and performance, and the finite element simulations are applied to verify the regulation performance of the designed structure for electromagnetic waves. Finally, the electromagnetic performance of the integrated dual-layer system is systematically analyzed.

### 2.1. Description of the Structure

**Figure 1:**
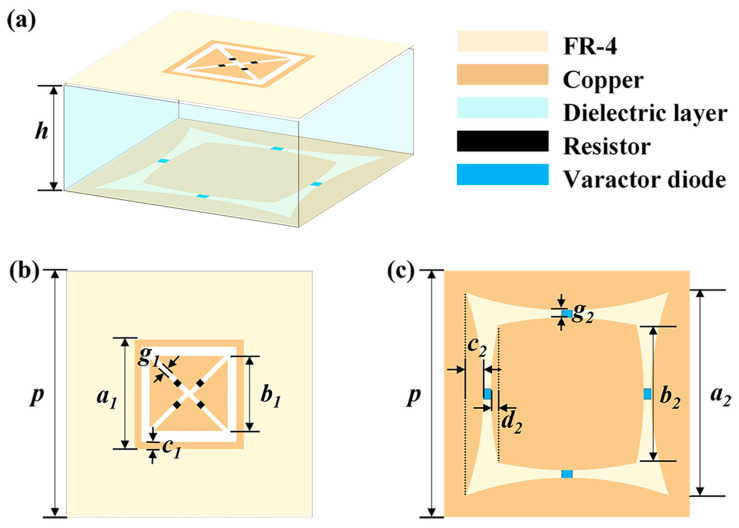
Schematic diagram of the FSSs. (**a**) Overall structure; (**b**) absorption layer unit; (**c**) tunable transmission layer unit.

In general, to design the multi-functional FSSs, a multi-layer structure needs to be adopted, where different layers will be separately responsible for absorption or transmission. As shown in Figure 1a, the proposed double-layer FSSs are composed of two cascaded metal pattern layers, as shown in dark brown. The light-yellow part represents dielectric substrate, which we use FR4 material with a thickness of 0.2 mm. A 30-mm-thick polymethacrylimide (PMI) layer is used as the support between the two layers of structure, as illustrated with light blue. Figure 1b,c details the structure of the absorption layer (top FSS layer) and tunable transmission layer (bottom TFSS layer) units, respectively, where the black sections indicate resistor locations, and the dark blue areas denote where varactor diodes are loaded. The various dimensional parameters in the figure are as follows: *p* = 40 mm; *h* = 30 mm; *a*_1_ = 17 mm; *b*_1_ = 12.6 mm; *c*_1_ = 1 mm; *g*_1_ = 0.91 mm; *a*_2_ = 36 mm; *b*_2_ = 24 mm; *g*_2_ = 1.5 mm; *c*_2_ = 3 mm; *d*_2_ = 1.5 mm; Resistor = 200 ohm.

### 2.2. Design of the Tunable Transmission Performance

**Figure 2:**
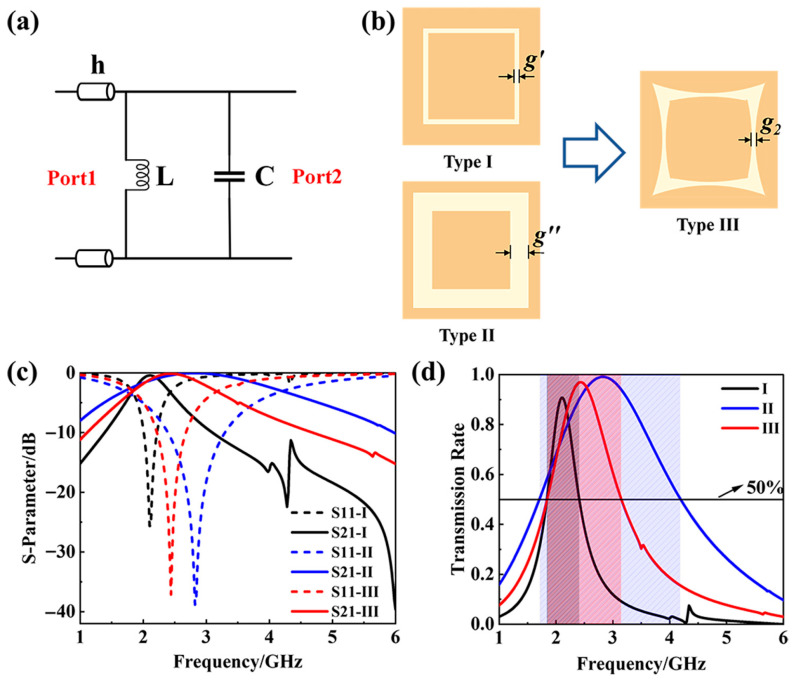
(**a**) L-C parallel bandpass filter circuit; (**b**) three types of the slotted metal patch; (**c**) S-parameter of the FSS using patterns in (**b**); (**d**) frequency bands covered by these three FSSs when their transmission rates exceed 50%.

In general, an L-C parallel circuit can function as a bandpass filter. The *ABCD* matrix [26,27] of the network in Figure 2a can be written as follows:

(1) $$\left[\begin{array}{cc} A & B\\ C & D \end{array}\right]=\left[\begin{array}{cc} 1 & 0\\ \frac{1}{j\omega L} & 1 \end{array}\right]\left[\begin{array}{cc} 1 & 0\\ j\omega C & 1 \end{array}\right]=\left[\begin{array}{cc} 1 & 0\\ j(\omega C-\frac{1}{\omega L}) & 1 \end{array}\right]$$

Using the conversion formula between *S* matrix and *ABCD* matrix, *S11* and *S21* can be written as follows:

(2) $$S11=\frac{-YZ_{0}}{2+YZ_{0}}$$

(3) $$S21=\frac{2}{2+YZ_{0}}$$

where $Y=j(\omega C-\frac{1}{\omega L})$, and *Z*_0_ is the characteristic impedance of the ports. If $\omega =1/\sqrt{LC}$, then, *Y* = 0, *S11* = 0, and *S21* = 1, which means complete transmission of electromagnetic waves.

Based on equivalent circuit theory, metallic gaps inherently exhibit capacitive characteristics, rendering slotted metal patch arrays equivalent to distributed capacitive networks. The capacitance is determined by the following formula:

(4) C≈ε0εrlwh,wh≪1

where *w* and *l*, respectively, represent the width and length of the gap, *ε*_0_ and *ε_r_* represent the dielectric constant of vacuum and the substrate, and *h* represents the thickness of the dielectric substrate.

This study advances beyond traditional straight-slot designs by introducing a novel gradient gap square-ring topology, as depicted in Figure 2b. Figure 2b illustrates three configurations: Type I and Type II represent conventional metal gap patches with fixed gaps of *g*′ = 1.5 mm and *g*″ = 6 mm, respectively. Type III constitutes a derivative design of these, incorporating *g*_2_ = *g*′ = 1.5 mm. The corresponding transmission characteristics for these three structures are presented in Figure 2c. Analysis of *S21* reveals that Type II exhibits the largest effective passband, followed by Type III. Furthermore, Figure 2d explicitly compares the frequency ranges where each structure achieves an electromagnetic wave transmission rate exceeding 50%. Compared to Type I, the asymmetric distribution of metal gaps along the curved slots in Type II and Type III establishes a gradient capacitance distribution. The tunability of transmission requires the loading of varactor diodes, so the gap value cannot be too large. Meanwhile, this gradient capacitance induces multi-mode coupling effects within the resonant region, ultimately resulting in an expanded broadband transmission window. Considering the comprehensive requirements of broadband transmission performance and tunability, Type III was ultimately selected as the final FSS unit.

**Figure 3:**
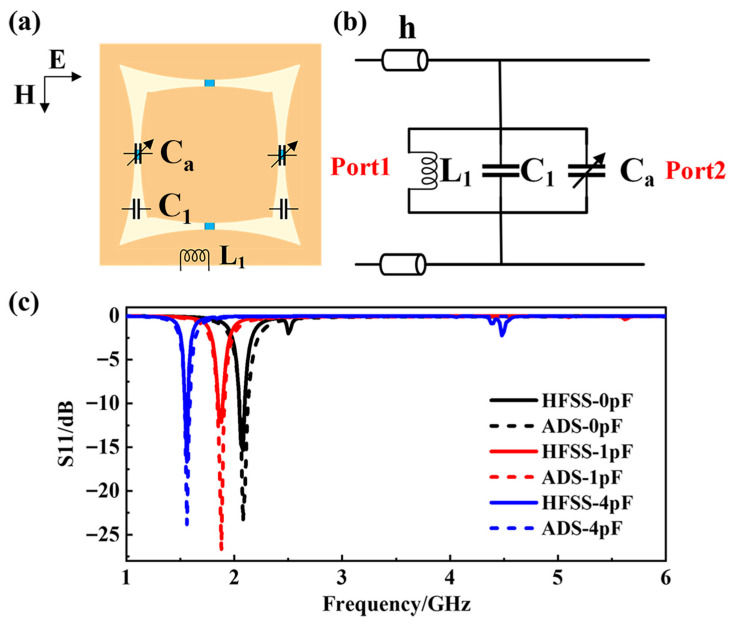
(**a**) Schematic diagram of tunable transmission layer; (**b**) equivalent circuit of tunable transmission layer; (**c**) comparison of simulation curves between ADS and HFSS.

To validate the design mechanism, an equivalent circuit model was established in Advanced Design System (ADS 2016.01) software. As shown in Figure 3a,b, inductor *L*_1_ characterizes the skin effect inductance of metallic strips, capacitor *C*_1_ represents the intrinsic distributed capacitance between arc-shaped slots, and *C_a_* corresponds to the equivalent value of the adjustable capacitance provided by the two external varactors. The electromagnetic characteristics of the tunable transmission layer were rigorously investigated through co-simulation using High Frequency Simulation Software (HFSS 2019) and ADS. A parametric study was conducted by configuring the varactor diodes with three discrete capacitance values of 0 pF, 1 pF, and 4 pF. Figure 3c comparatively presents the simulated *S11* parameters from both platforms, with capacitance labels explicitly corresponding to HFSS configurations. In ADS implementations, the 0 pF in HFSS setting equates to disabling the varactor branch, i.e., open-circuit condition, under which situation *L*_1_ = 1.577 nH and *C*_1_ = 3.685 pF. Subsequently, the value of varactor diodes in HFSS were set to 1 pF and 4 pF successively, which are equivalent to the *C_a_* values in ADS of 0.88 pF and 2.88 pF, respectively, demonstrating parameter correlation between finite element simulation and equivalent circuit modeling.

The analysis reveals a consistent downward shift in resonant frequency from 2.08 GHz to 1.56 GHz as capacitance increases, confirming the structure’s frequency agility through varactor tuning. Furthermore, both simulation platforms exhibit *S11* convergence to 0 dB above 2.5 GHz, indicating that the structure has a near-perfect reflection effect for electromagnetic waves at higher frequencies. This bi-modal behavior substantiates the design’s capability for adaptive electromagnetic control, combining tunable low-frequency transmission with inherent high-frequency harmonic suppression.

### 2.3. Design of the Absorption Performance

**Figure 4:**
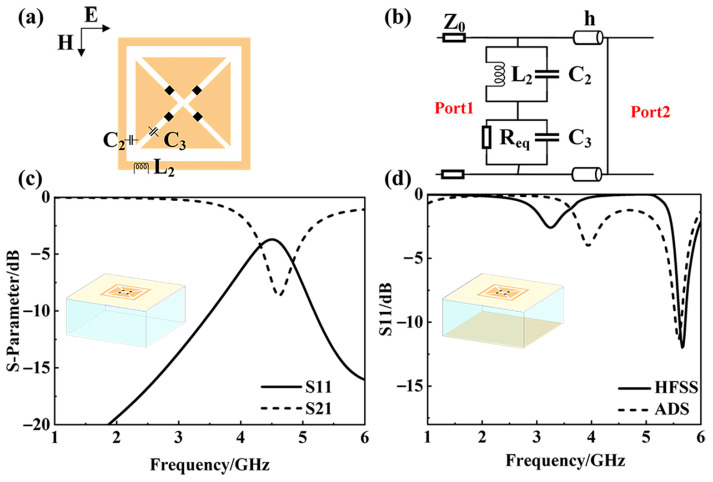
(**a**) Schematic diagram of absorption layer; (**b**) equivalent circuit of absorption layer; (**c**) S-parameter curves of the absorption layer; (**d**) comparison of *S11* curves in HFSS and ADS for the absorption layer with a metal plate added.

By employing square-ring-cross-slot topology, a lossy structure is designed as the passive absorber layer. Figure 4a illustrates the schematic of the passive layer unit cell in the dual-layer configuration. Leveraging bandpass filter theory and equivalent transmission line principles, the unit cell dimensions are minimized to shift the passband toward lower frequencies. Broadband impedance matching and reduced insertion loss are achieved through lumped resistors symmetrically integrated along the patch slots. In Figure 4b, an equivalent circuit of the absorber layer is established, where *L*_2_ represents the equivalent inductance of the square ring, *C*_2_ represents the coupling capacitance between the square ring and the inner patch, and the capacitance between the cross-shaped slits is represented by *C*_3_. For the four symmetrically distributed resistors, their equivalent value is replaced by *R_eq_*. Therefore, the input impedance *Z_in_* of this absorber unit cell can be obtained:

(5) $$Z_{in}=Z_{L_{2}||C_{2}}+Z_{R_{eq}||C_{3}}=\frac{1}{\frac{1}{j\omega L_{2}}+j\omega C_{2}}+\frac{1}{\frac{1}{R_{eq}}+j\omega C_{3}}$$

Perfect matching to free-space impedance *Z*_0_ requires *Z_in_* = *Z*_0_. The integrated resistor *R_eq_* is critical for simultaneously satisfying real part matching and energy dissipation over a bandwidth. Through the curve fitting in Figure 4d, the equivalent circuit parameters can be obtained as follows: *L*_2_ = 2.8575 nH, *C*_2_ = 0.459 pF, *C*_3_ = 2.5549 pF, *R_eq_* = 356.5 ohm.

As expected, the absorption layer allows the incident waves to transmit to the tunable transmission layer at a lower frequency, while having a lossy effect on higher-frequency incident waves. This dual-function feature is depicted in Figure 4, which shows the S-parameter curves under two situations. In Figure 4c, *S21* is close to 0 dB below 2.5 GHz frequencies, indicating that the absorber layer alone has a complete transmission effect in this band. Simultaneously, it is obvious that *S21* decreases in the higher-frequency band, which needs further research. Setting the bottom layer as the metal plate, Figure 4d shows the simulated *S11* curves of the composite structure from both HFSS and ADS software. Both simulations reveal a resonance point between 5 GHz and 6 GHz, where the structure demonstrates strong absorption.

Separate modeling of the absorption layer and subsequent simulation analysis of the structure stacked onto a metal plate have verified its transmissive behavior towards lower-frequency electromagnetic waves and its absorptive property at higher frequencies. This verification provides a foundation for the subsequent design of the double-layer architecture.

### 2.4. Integrated Structure and Simulation Results

This section will systematically study the dynamic response characteristics of the bi-layer composite structure under different regulation parameters through electromagnetic simulation methods.

**Figure 5:**
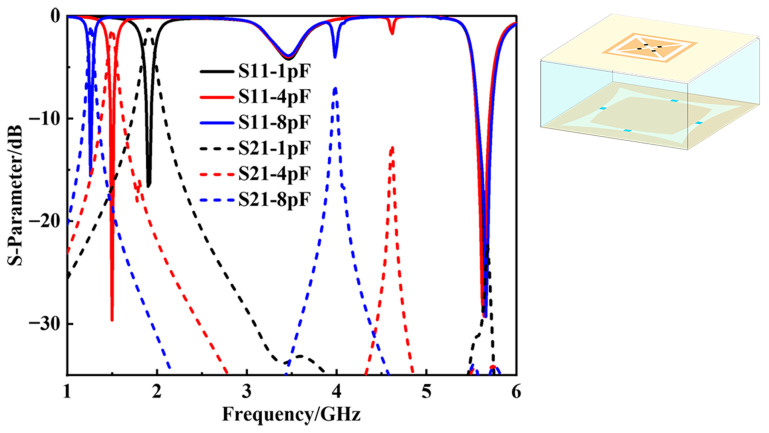
*S11* and *S21* curves with different capacitance values.

A composite structure consisting of an absorber layer and a tunable transmission layer was established. The absorption layer is positioned above the tunable one, separated by the PMI dielectric layer. Using capacitance as a variable parameter to study the electromagnetic wave regulation effect, Figure 5 shows the simulation results. In the lower-frequency band, as capacitance increases from 1 pF to 8 pF, the electromagnetic transmission window shifts from 1.9 GHz to around 1.26 GHz with *S21* approaching −1 dB. Meanwhile, in the higher-frequency range, all three capacitance values exhibit extremely low *S21* values and similar *S11* curves. Specifically speaking, the *S11* value is below −10 dB within the range of 5.56 GHz to 5.72 GHz, and a resonance point occurs at 5.65 GHz, where *S11* approaches −30 dB.

**Figure 6:**
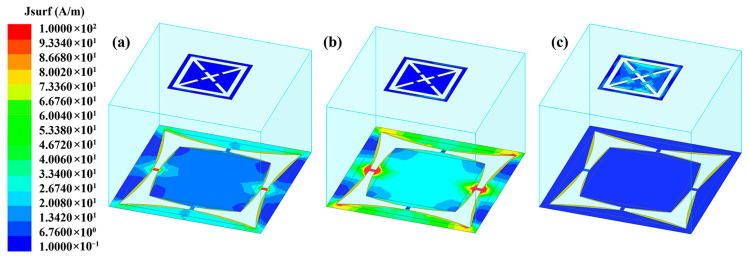
Surface current distribution diagrams of (**a**) capacitance being 4 pF at 1.5 GHz, (**b**) capacitance being 8 pF at 1.26 GHz, and (**c**) capacitance being 4 pF at 5.65 GHz.

To investigate the role of unit structure in overall functionality, surface current density distributions at different frequencies were analyzed. Figure 6a,b presents the surface current distributions at resonant frequencies of 1.5 GHz and 1.26 GHz corresponding to varactor capacitances of 4 pF and 8 pF, respectively. The comparative analysis reveals concentrated current patterns predominantly localized within the tunable layer under both conditions. Notably, the 8 pF configuration exhibits intensified current density near the varactor diodes, with a maximum magnitude of 450 A/m vs. 156 A/m at 4 pF, demonstrating enhanced capacitive coupling effects and highlighting the varactor diodes’ critical role in lower-frequency transmission band regulation.

Figure 6c displays the surface current distribution at 5.65 GHz under the state of loading 4 pF capacitance, corresponding to the absorption resonance frequency identified in Figure 4. Distinct from the transmission-mode current patterns in Figure 6a, this absorption-dominant configuration demonstrates significantly different current localization characteristics, with concentrated current flows predominantly confined within the absorption layer. The observed operational mode transition that forms broadband electromagnetic transmission to wave absorption at higher frequencies substantiates the structure’s dual-function capability that enables dynamic regulation of the transmission window and solidification of the absorption frequency band through optimized interlayer coupling.

## 3. Experimental Verification

### 3.1. Prototype and Measurement Setup

**Figure 7:**
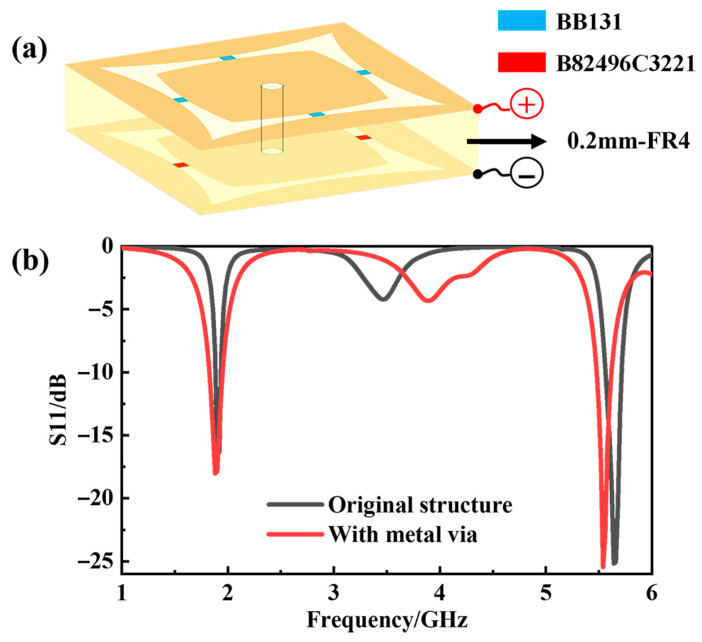
(**a**) Three-dimensional view of feeding setup for the tunable FSS layer; (**b**) comparison of *S11* curves between the initial design and the structure with metal via added.

Due to the need to activate the varactor diodes on the second layer, the prepared samples differ somewhat from the simulation model. As depicted in Figure 7a, on the underside of the transmission layer’s FR4, the same structure as the upper side was printed, and two 220 nH inductors (B82496C3221), which were drawn in red, were soldered in the middle of the arc-shaped slot, allowing the DC bias voltage of the varactor diodes to pass. A circular hole with a diameter of 0.3 mm was opened at the center of the intermediate metal patch to ensure the feedline could conduct. The positive and negative poles of the bias voltage of the varactor diode are loaded on respective sides of the tunable layer. To evaluate the impact of the through-hole feeding configuration on the structure’s performance, the design shown in Figure 7a was simulated in HFSS. With the capacitance value fixed at 1 pF and other parameters unchanged, Figure 7b compares the *S11* curves of the original design and the modified design incorporating the double-sided metal vias. The red curve in Figure 7b corresponds to the geometry to include the two-sided FSS plus the via in Figure 7a. The results show that the reflection coefficient exhibits minute change after adding the vias, demonstrating the feasibility of the through-hole feeding approach. The difference around 5.5 GHz is mainly caused by the coupling capacitance between the double-sided FSS. Under the condition that the FR4 thickness remains unchanged, the higher the frequency, the greater the equivalent coupling capacitance. To reduce the difference, the thickness of the FR4 layer can be reduced as much as possible, but at the same time, the actual material conditions need to be taken into consideration.

**Figure 8:**
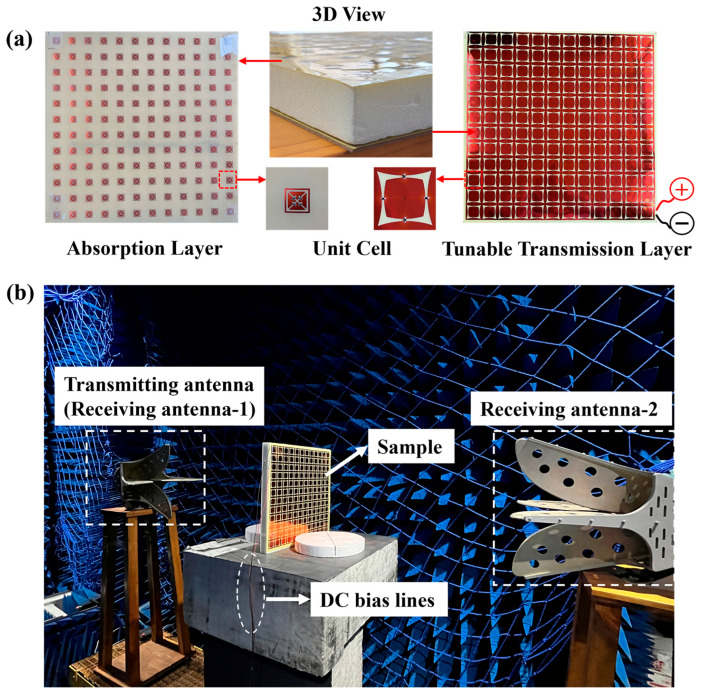
(**a**) Photograph of the fabricated prototype; (**b**) photograph of the measurement setup.

Based on the above design, a dual-layer frequency selective surface with a size of 500 mm × 500 mm was fabricated. Figure 8a shows the three-dimensional view of the test sample, the overall view of the two FSS layers, and the detailed structure of the unit. Two layers of FSSs were made using standard printed circuit board (PCB) technology, with the dielectric substrate using 0.2-mm-thick FR4, as well as a 30-mm-thick PMI for support material. The reflection coefficient and transmission coefficient of the FSS were measured in an anechoic chamber using the free-space measurement method. During the free-space measurement, a pair of broadband dual-ridge horn antennas connected to a vector network analyzer (Keysight, George Town, Malaysia) were used as transmitting and receiving ends. Figure 8b is a photograph of the test site, with the transmitting antenna facing the absorption layer of the FSSs to measure the *S11* parameter. On the other side of the FSSs is the receiving antenna, used to measure the *S21* parameter.

### 3.2. Experimental Results

**Figure 9:**
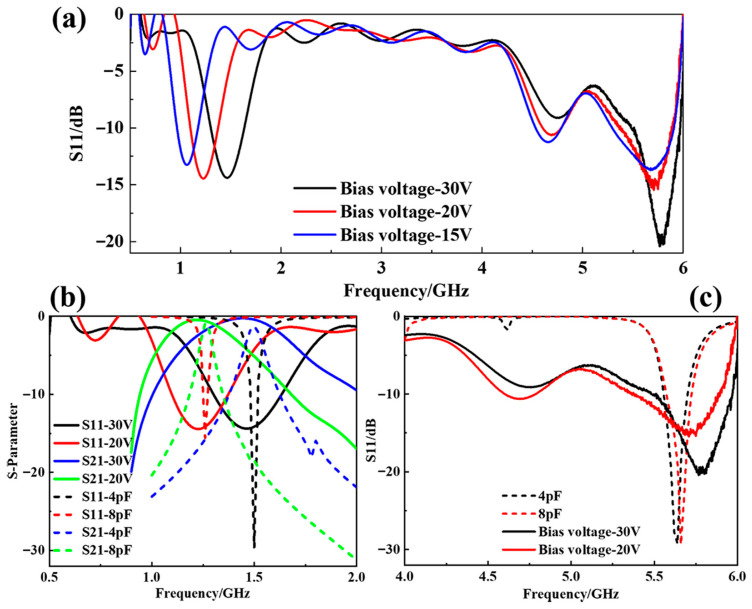
Measured results. (**a**) Reflectivity curves of the tunable FSSs within the 0.5–6 GHz frequency band under different voltage excitations; (**b**) comparison of simulation and measured results of *S11* and *S21* at a lower-frequency band; (**c**) comparison of simulation and measured results of *S11* at a higher-frequency band.

The varactor diode BB131 (illustrated in Figure 7a) from NXP Inc. (Eindhoven, The Netherlands), featuring excellent linearity, can provide a tunable range of capacitance from 1 pF to 10 pF for a reverse bias voltage between 30 and 1.5 V. Figure 9a presents the *S11* characteristics of the tunable FSSs under three distinct reverse bias voltage levels. The varactor diodes present lower capacitance at a large reverse bias voltage, and higher capacitance at a small voltage. As the bias voltage decreases from 30 V down to 15 V, the resonant frequency at the lower band shifts progressively from 1.5 GHz to 1.02 GHz. It indicates that the lower-frequency transmission window of the dual-layer FSS structure can be adjusted by regulating the bias voltage on the varactor diode, while maintaining the higher-frequency absorption effect. Figure 9b compares simulated and measured *S11* and *S21* responses across the 0.5–2 GHz frequency range, where the dotted lines represent the simulation results and the experimental curves are illustrated by solid lines. Two resonant peaks near 1.5 GHz and 1.26 GHz were selected as the analysis objects. It can be found that the values of *S11* and *S21* at the resonant frequency points corresponding to the measured and simulation results have good agreement. Figure 9c details the simulated and measured *S11* comparison in the 4–6 GHz range. According to the measured results, it can be found that the absorption performance in the higher-frequency band remains unchanged, and there is a strong absorption peak near 5.65 GHz, which is basically consistent with the law of the simulation results.

The small differences between the simulated and measured results may come from fabrication tolerances and the finite size of the prototype. Slight differences in the position of the absorption peaks exist that result from the variation in junction resistance and package inductance of the varactor diodes. There is a difference in the width between the calculated and measured results. On the one hand, this is because the theoretical simulation is an infinitely ideal size, while the measured sample is of a finite size. On the other hand, it is caused by fabrication tolerances and systematic errors of the measurement setup. However, the comprehensive simulation and experimental validation conclusively demonstrate the dual-functional capabilities of the proposed tunable dual-layer FSS structure.

## 4. Discussion

Table 1 presents a comparison of the structure and performance between this work and previous related studies. In general, while maintaining the absorption performance, the tunable structure proposed in this research can achieve a wide tunable range within the low-frequency range, and the insertion loss (IL) is sufficiently low, with the overall range from 0.26 dB to 1.41 dB.

## 5. Conclusions

This paper proposes a compact dual-layer FSS architecture achieving dual-band electromagnetic control through coordinated layer functionality. The architecture achieves electrically tunable transmission characteristics in the L-band, exhibiting a remarkable 64% frequency tuning range from 1.26 GHz to 1.9 GHz through varactor capacitance adjustment, and the experimental results are in line with the simulation. Simultaneously, it maintains significant isolation between operational states, ensuring reliable mode differentiation. For higher-frequency operation, the structure transitions to an absorption-dominant regime between 5.5 and 6 GHz, where measured results confirm a consistent −10 dB reflection bandwidth exceeding 300 MHz with peak absorption efficiency of 97% at 5.65 GHz. The architecture’s decoupled control mechanisms address critical needs for multifunctional electromagnetic materials, particularly suggesting strong potential for practical implementation in next-generation wireless communication and radar systems. Controlling the switching time of the varactors with pulse signals can further optimize the regulation characteristics of the FSS. In the future, this design can further optimize the absorption bandwidth, thus having better value for practical applications in the fields of radome and multi-antenna communication.

## Figures and Tables

**Table 1:** Performance comparison with previous tunable FSSs.

Reference	Absorption	Transmission Tunable	TR ^1^ (GHz)	IL ^2^ (dB)	Thickness ^3^
[26]	Yes	No	7.4–12.1	1.5	0.13*λ_L_*
[27]	No	No	6.92–13.02	0.43–3	0.114*λ_L_*
[11]	No	Yes	3.8–5.2	0.59	0.107*λ_L_*
[28]	No	Yes	2.92–4.66	4.5–1	0.122*λ_L_*
[29]	No	Yes	1.14–1.35 and 2.01–2.61	N.A. ^4^	0.006*λ_L_*
[30]	No	Yes	1.8–4.5	1.2–3.9	0.04*λ_L_*
[15]	No	Yes	3–4.55	2.48	0.005*λ_L_*
This work	Yes	Yes	1.26–1.9	0.26–1.41	0.103*λ_L_*

^1^ Tunable range of the implementation band. ^2^ Insertion loss. ^3^ *λ_L_* = free space wavelength at lowest operating frequency. ^4^ No specific values.

## Data Availability

The original contributions presented in this study are included in the article. Further inquiries can be directed to the corresponding authors.
